# Supplementary material for: Driving factors in pediatric emergency department use: an ecological retrospective study
Source: PLoS One. 2026 May 28;21(5):e0344153. doi: 10.1371/journal.pone.0344153 (PMC13218463; doi:10.1371/journal.pone.0344153)
Supplement: Attachment — Submitted filename: reviewer_JNS.docx [file pone.0344153.s003.docx]

# Journal Requirements

When submitting your revision, we need you to address these additional requirements.

1. Please ensure that your manuscript meets PLOS ONE's style requirements, including those for file naming. The PLOS ONE style templates can be found at <https://journals.plos.org/plosone/s/file?id=wjVg/PLOSOne_formatting_sample_main_body.pdf> and <https://journals.plos.org/plosone/s/file?id=ba62/PLOSOne_formatting_sample_title_authors_affiliations.pdf>

A: We have revised the manuscript and source files to conform to PLOS ONE’s formatting and file-naming requirements, following the journal’s style templates.

2. Your ethics statement should only appear in the Methods section of your manuscript. If your ethics statement is written in any section besides the Methods, please delete it from any other section.

A: We have removed the ethics statement from the Acknowledgments. It now appears only in the Methods section, as requested.

3. We note that you have indicated that there are restrictions to data sharing for this study. For studies involving human research participant data or other sensitive data, we encourage authors to share de-identified or anonymized data. However, when data cannot be publicly shared for ethical reasons, we allow authors to make their data sets available upon request. For information on unacceptable data access restrictions, please see <http://journals.plos.org/plosone/s/data-availability#loc-unacceptable-data-access-restrictions>.

Before we proceed with your manuscript, please address the following prompts:

a) If there are ethical or legal restrictions on sharing a de-identified data set, please explain them in detail (e.g., data contain potentially identifying or sensitive patient information, data are owned by a third-party organization, etc.) and who has imposed them (e.g., a Research Ethics Committee or Institutional Review Board, etc.). Please also provide contact information for a data access committee, ethics committee, or other institutional body to which data requests may be sent.

A: The dataset consists of administrative records for all patients attending the pediatric emergency department over a two-year period. Use of these data is irrespective of individual informed consent and was authorized because the project was classified as a quality-of-care study by the Geneva Research Ethics Commission and the hospital’s institutional review board.

Access to the raw data was granted to us in our capacity as hospital staff. Because not all patients provided consent for use of their data, the Geneva Research Ethics Commission and the institutional board of the University Hospitals of Geneva do not permit public sharing of the original dataset or of derived datasets.

Requests for access to deidentified data must be reviewed and approved by the Geneva Research Ethics Commission (https://submissions.swissethics.ch/en), with reference to the request number given in the article: req-2025-00269. In addition, the University Hospitals of Geneva require a signed Data Transfer and Use Agreement and a Data Transfer and Processing Agreement before patient data can be shared with third parties. After these approvals, data would be provided by the authors.

We have updated the Data Availability statement to read: “*Deidentified data will be made available upon reasonable request, conditional on approval of the protocol by the Geneva Research Ethics Commission (https://submissions.swissethics.ch/en, request number req-2025-00269) and on the signature of a Data Transfer and Use Agreement and a Data Transfer and Processing Agreement with the University Hospitals of Geneva.*”

b) If there are no restrictions, please upload the minimal anonymized data set necessary to replicate your study findings to a stable, public repository and provide us with the relevant URLs, DOIs, or accession numbers. Please see <http://www.bmj.com/content/340/bmj.c181.long> for guidelines on how to de-identify and prepare clinical data for publication. For a list of recommended repositories, please see <https://journals.plos.org/plosone/s/recommended-repositories>. You also have the option of uploading the data as Supporting Information files, but we would recommend depositing data directly to a data repository if possible. Please update your Data Availability statement in the submission form accordingly.

A: As detailed under point 3a, the authors are not authorized to deposit the dataset in a public repository, even in deidentified form, because the data were collected and analyzed irrespective of individual patient consent and the ethics and institutional approvals do not allow public data sharing.

4. In the online submission form, you indicated that data will be made available upon request, conditioned to the approval of a proposal with a signed data access agreement and to the approval of the ethical commission concerned.

All PLOS journals now require all data underlying the findings described in their manuscript to be freely available to other researchers, either 1. In a public repository, 2. Within the manuscript itself, or 3. Uploaded as supplementary information.
This policy applies to all data except where public deposition would breach compliance with the protocol approved by your research ethics board. If your data cannot be made publicly available for ethical or legal reasons (e.g., public availability would compromise patient privacy), please explain your reasons on resubmission and your exemption request will be escalated for approval.

 A: As noted under point 3a, this study is based on routinely collected administrative data for all PED attendances, irrespective of patient consent, and was approved by the Geneva Research Ethics Commission and the hospital ethics board as a quality-of-care study. Access to the data was granted only to hospital staff.

Because consent was not obtained, making the data publicly available, even in deidentified form, would not comply with the protocol approved by the ethics committee and the institutional data protection rules. Data therefore cannot be deposited in a public repository for ethical and legal reasons. As described in 3a, deidentified data may be made available upon request, subject to ethics approval and appropriate data transfer agreements with the University Hospitals of Geneva.

5. We note that Figure 1, S2, in your submission contain [map/satellite] images which may be copyrighted. All PLOS content is published under the Creative Commons Attribution License (CC BY 4.0), which means that the manuscript, images, and Supporting Information files will be freely available online, and any third party is permitted to access, download, copy, distribute, and use these materials in any way, even commercially, with proper attribution. For these reasons, we cannot publish previously copyrighted maps or satellite images created using proprietary data, such as Google software (Google Maps, Street View, and Earth). For more information, see our copyright guidelines: http://journals.plos.org/plosone/s/licenses-and-copyright.

We require you to either (1) present written permission from the copyright holder to publish these figures specifically under the CC BY 4.0 license, or (2) remove the figures from your submission:

a. You may seek permission from the original copyright holder of Figures 1, S2, to publish the content specifically under the CC BY 4.0 license.

We recommend that you contact the original copyright holder with the Content Permission Form (http://journals.plos.org/plosone/s/file?id=7c09/content-permission-form.pdf) and the following text: “I request permission for the open-access journal PLOS ONE to publish XXX under the Creative Commons Attribution License (CCAL) CC BY 4.0 (http://creativecommons.org/licenses/by/4.0/). Please be aware that this license allows unrestricted use and distribution, even commercially, by third parties. Please reply and provide explicit written permission to publish XXX under a CC BY license and complete the attached form.” Please upload the completed Content Permission Form or other proof of granted permissions as an "Other" file with your submission. In the figure caption of the copyrighted figure, please include the following text: “Reprinted from [ref] under a CC BY license, with permission from [name of publisher], original copyright [original copyright year].”

b. If you are unable to obtain permission from the original copyright holder to publish these figures under the CC BY 4.0 license or if the copyright holder’s requirements are incompatible with the CC BY 4.0 license, please either i) remove the figure or ii) supply a replacement figure that complies with the CC BY 4.0 license. Please check copyright information on all replacement figures and update the figure caption with source information. If applicable, please specify in the figure caption text when a figure is similar but not identical to the original image and is therefore for illustrative purposes only.

The following resources for replacing copyrighted map figures may be helpful: USGS National Map Viewer (public domain): <http://viewer.nationalmap.gov/viewer/>, The Gateway to Astronaut Photography of Earth (public domain): <http://eol.jsc.nasa.gov/sseop/clickmap/>, Maps at the CIA (public domain): <https://www.cia.gov/library/publications/the-world-factbook/index.html>,and <https://www.cia.gov/library/publications/cia-maps-publications/index.html>, NASA Earth Observatory (public domain): <http://earthobservatory.nasa.gov/>, Landsat: <http://landsat.visibleearth.nasa.gov/>, USGS EROS (Earth Resources Observatory and Science (EROS) Center) (public domain): [http://eros.usgs.gov/#](http://eros.usgs.gov/), Natural Earth (public domain): <http://www.naturalearthdata.com/>

A: Figures 1 and S2 do not contain copyrighted satellite imagery or proprietary map tiles. All color fills and overlays correspond to our study data. The maps are based on the official neighborhood boundaries and population statistics provided as open data by the Geneva State Geomatics Services (SITG): available at https://sitg.ge.ch/donnees/ocs-population-ssecteur

The outlines of the lake and rivers were derived from OpenStreetMap geometry and redrawn for this figure; no original OpenStreetMap tiles or images are reproduced. We have updated the method section and the figure captions to clarify the sources.

6. Please include captions for your Supporting Information files at the end of your manuscript, and update any in-text citations to match accordingly. Please see our Supporting Information guidelines for more information: http://journals.plos.org/plosone/s/supporting-information.

A: We have added captions for all Supporting Information files at the end of the manuscript and checked that all in-text citations correspond to the correct Supporting Information items. In addition, to clarify the origin of the spatial data used in the maps, we added a Methods subsection entitled “Spatial data”:

*2.3	Spatial Data*

*All spatial and population data used in the study are based on official population statistics and the delimitation of Geneva neighborhoods as provided openly by the Geneva State Geomatics Services^19^.*

*^19^ Statistique cantonale de la population (information au sous-secteur) | Catalogue SITG.* [*https://sitg.ge.ch/donnees/ocs-population-ssecteur*](https://sitg.ge.ch/donnees/ocs-population-ssecteur)

and updated the figure captions to specify that the outlines of rivers and the lake were obtained from OpenStreetMap.

7. If the reviewer comments include a recommendation to cite specific previously published works, please review and evaluate these publications to determine whether they are relevant and should be cited. There is no requirement to cite these works unless the editor has indicated otherwise.

A: We thank the editor for their suggestions. We have evaluated the recommended publications and cited those that we considered relevant and consistent with the scope of our manuscript.

# Reviewer #1

1. This manuscript represents a scientifically rigorous investigation, providing a clear and well-substantiated analysis of spatial variation in pediatric emergency department (PED) utilisation. The findings demonstrate that low-acuity visits are strongly modulated by socio-economic vulnerability and proximity to the hospital, whereas high-acuity visits remain largely unaffected by contextual factors. Notable strengths include the comprehensive two-year dataset, high-resolution geospatial and socio-demographic information, standardised CTAS triage, and the application of fully adjusted mixed-effects models. The limitations, including the retrospective ecological design, absence of individual-level health data, and restriction to a single urban centre, are appropriately acknowledged.

A: We sincerely thank the reviewer for this very positive assessment. We are pleased that the strengths of the study, as well as the balance between findings and limitations, were clearly conveyed.

2. To further enhance the manuscript, the authors should elaborate on the mechanistic pathways underlying the impact of socio-economic vulnerability, contextualise the findings relative to healthcare systems in other high-income settings, provide a summary table or figure of primary effects, clearly articulate policy implications, and explicitly delineate the novel contributions of this study in the context of existing literature

A: We thank the reviewer for these constructive suggestions. We have substantially revised the Discussion to address each point as follows:

*Main findings and interpretation*: we complemented this subsection that summarizes the main adjusted effects of distance, socio-economic vulnerability and pediatrician density across CTAS levels, as depicted in Figure 2 and Tables 2 and 3, to make the primary effects easier to interpret.

*Mechanistic pathways*: We added a dedicated subsection (Section 4.1.4) that links neighborhood socio-economic vulnerability to low-acuity PED use, distinguishing structural, behavioral and informational mechanisms and integrating recent empirical and conceptual work.

*4.1.5	Comparison with other high-income systems*: We introduced a subsection (Section 4.1.5) that situates our findings in relation to evidence from other high-income, universal or near-universal health systems and to prior work on distance-related “distance decay” effects.

*Policy implications*: We expanded and reorganized the policy-focused part of the Discussion (Section 4.2) to clarify the implications of our findings, with explicit reference to literature on interventions targeting non-urgent pediatric ED use and on integrated, multi-component approaches.

*Novel contributions and future research*: We added a new subsection (Section 4.3) that explicitly summarizes how our study extends existing work (joint modeling of distance, neighborhood vulnerability and pediatrician density by acuity) and briefly outlines future directions, including applying similar geospatial modeling to multicenter datasets and evaluating the role of digital triage tools.

New references were added to support the different new subsections. We hope the discussion now meets the reviewer expectations.

# Reviewer #2

1. Authors must clearly state in Discussion and Conclusion that no individual-level inference can be drawn.

A: We agree and have clarified this in the Limitations section, which now reads:
“*Second, the ecological design does not allow inference at the individual level and introduces potential ecological bias.*”

2. Rewrite interpretations to reflect neighborhood-level associations only.

A: We have revised the Discussion to consistently express results at neighborhood level. For example, we now state:

*“Proximity was a key determinant of neighborhood-level PED use. Neighborhoods within 5 km of the hospital had higher PED use, especially […]”*

*“Socio-economic vulnerability was a strong, independent predictor of* *neighborhood-level PED use, with […]”*

*“Primary care pediatrician density had a limited but measurable inverse association with neighborhood-level PED use, observed only […]”*

*“Proximity alone accounted up to 15% of neighbourhood level visits, particularly […]”*

3. Why was centroid distance preferred over population-weighted centroids or street-network distance?

A: We did not have access to the within-neighborhood distribution of the population and therefore could not compute population-weighted centroids. Street-network distance would have been a reasonable alternative, but was not implemented at the time of the study. We chose straight-line (crow-fly) distance from the neighborhood centroid because neighborhoods are relatively small and numerous, and because the main effect of interest was a short-range gradient centered on a central, compact urban location (the PED).

We have added the following to the Limitations:

*“Third, the measure considered for the distance from neighborhood to PED are the straight-line distances from the neighborhood centroid, which may not be perfectly representative of the actual travel time and thus of the associated burden.”*

4. If unique patients are used, explain whether a child with multiple visits counts once per CTAS category or once overall.

A: We thank the reviewer for this point. A child with multiple visits can contribute once to each CTAS category in which they were seen. The Methods now read:

*The primary outcome was the neighborhood-level incidence of PED use per triage level, defined as the number of unique pediatric patients per 100 children aged 0-16 in the neighborhood population. If a child had multiple visits at different triage level, he counted one for each of the triage level concerned.*

5. Clarify whether distance was measured “as the crow flies” or using travel time / road network.

A: We have clarified this in the Methods as follows:

*Distance to PED was measured as the straight-line distance from the geographic centroid of each neighborhood to the PED.*

6. Typographical errors:

“Halving diatnce” → “Halving distance”. “Conterfactual” → “Counterfactual”.

A: We thank the reviewer for the careful reading and have corrected the typos: “Halving diatnce” to “Halving distance” and “Conterfactual” to “Counterfactual”.

7. “Geneva resident= 1 (%)” is unclear

A: We have modified this label to “Living in Geneva state (%)” for clarity.

8. Table 1 mixes visits and patients; columns unclear.

A: We agree that mixing visits and patients in a single table was confusing. We have separated the information into two tables: Table 1a (visits) and Table 1b (unique patients). In Table 1b we added the note:

*“A patient attending several times the PED during the period for various CTAS level is counted in each CTAS level. As a consequence, the sum of all CTAS levels is superior to the Overall column.”
We modified the results to refer to both tables.*

We have updated the Results section to refer to both tables accordingly.

Sincerely Yours,

Denis Mongin,

On behalf of all coauthors
