# Supplementary material for: Driving factors in pediatric emergency department use: an ecological retrospective study
Source: PLoS One. 2026 May 28;21(5):e0344153. doi: 10.1371/journal.pone.0344153 (PMC13218463; doi:10.1371/journal.pone.0344153)
Supplement: Attachment — Submitted filename: reviewer.docx [file pone.0344153.s004.docx]

Reviewer #1: Thank you for the opportunity to review this manuscript. The authors have adequately addressed the points raised in my previous comments, and I find the revisions satisfactory.

Authors: we thank the reviewer for its positive feedback.

Reviewer #3: As the statistical reviewer I will focus on methods and reporting. note that this is the first time I see this paper. The methods are well described and appropriate. The key limitations, including ecological bias, are discussed. perhaps be more careful when you imply individual level inference at times.

Authors: We thank the reviewer for its positive feedback.

Major

1) use an appropriate research checklist, e.g. STROBE probably in this case.

Authors: We completed the STROBE checklist and uploaded it along the manuscript.

2) explain if all the data were complete for the analyses, are they likely are at this level.

Authors:
Data were complete, as they are aggregated data and administrative data.
We added to the result, section 3.2:
*“No missing data were present in the aggregated data.”*

Minor

1) How was pediatrician density operationalised

Authors: Thank you for raising this point. The operationalization of pediatrician density was described in the Methods section under 'Exposures of interest.' Specifically, we retrieved the addresses of all non-hospital pediatricians practicing in the Canton of Geneva from the Swiss Federal Register of Medical Professions [22]. Each address was then geocoded using the state geocoding API [20] to obtain precise geographic coordinates. Rather than computing a crude density per neighborhood, we adopted a spatial smoothing approach: for each neighborhood, we counted all pediatricians located within a fixed 2 km radius of the neighborhood centroid and expressed this as a density per 1,000 children. This radius-based approach is more representative of the actual availability of pediatricians from a family's perspective, as it captures the nearby supply of providers regardless of whether they happen to fall within the same administrative unit. We have reviewed and complemented this paragraph which now reads:

“*Pediatrician density was derived from the Swiss Federal Register of Medical Professions [22] from which the addresses of all non-hospital pediatricians practicing in the Canton of Geneva were extracted. Each address was geocoded using the state geocoding API [20] to obtain precise geographic coordinates. For each neighborhood, the number of non-hospital pediatricians located within a fixed 2 km radius of the neighborhood centroid was counted and expressed per 1,000 children. This radius-based measure reflects the availability of pediatric primary care from a family's perspective, as it captures the nearby supply of providers irrespective of administrative boundaries**.”*

2) how was the NSVI index validated?

Authors: The NSVI has not undergone formal psychometric validation. It is a composite score developed by the “Centre d'analyse territorial des inégalités à Genève” (CATI-GE), a joint university–state institute dedicated to studying socio-economic inequalities in Geneva. The index is used operationally by cantonal authorities to allocate targeted funding to deprived neighborhoods, providing a degree of face and content validity. It has also been used in peer-reviewed research, including a study by our group on access to COVID-19 testing and outcomes, where it showed strong associations with both testing uptake and disease severity (Mongin et al., eClinicalMedicine 2022; doi:10.1016/j.eclinm.2022.101352). We have added a brief note in the Methods section acknowledging that the NSVI has not been formally validated but has demonstrated consistent associations with health outcomes in prior work:

“*Socio-economic Status was assessed using the Neighborhood Socio-economic Vulnerability Index (NSVI), a composite score developed by the Centre d'analyse territorial des inégalités à Genève (CATI-GE), a joint university–state institute dedicated to studying socio-economic inequalities in Geneva [21], The NSVI ranges from 0 to 6 and is constructed from state-provided indicators including income, unemployment, and social benefits (full criteria are provided in Supplementary Table 1). Although the NSVI has not undergone formal psychometric validation, it is used operationally by cantonal authorities to guide the allocation of targeted funding to deprived neighborhoods, supporting its face and content validity. The index has also demonstrated consistent associations with health outcomes in prior peer-reviewed research, including a study conducted by our group in which higher NSVI scores were associated with reduced access to COVID-19 testing and worse disease outcomes [22].”*

3) I'm not sure the "simulations" should be called that - they are model derived predictions under the same assumptions and "all else being equal". other than that, it is an appropriate approach.

Authors: We agree with the reviewer and have replaced ''counterfactual simulations' with 'counterfactual estimation’ throughout the manuscript to better reflect that these are derived from the fitted model under hypothetical conditions, holding all other factors constant.

4) perhaps also examine moran's I on the random effects as well, not only the residuals.

Thank you for this suggestion. We computed Moran's I on the estimated postal-code random intercepts for each CTAS level. Values were non-significant for CTAS 1 and CTAS 2, but significant for CTAS 3 (0.23, p<0.001) and CTAS 4* (0.16, p<0.05). This indicates that, for lower-acuity visits, some broad-scale spatial structure at the postal-code level remains unexplained by our exposure variables. This is not unexpected: while the exponential distance function captures the sharp, short-range proximity effect, broader geographic pattern, such as differences in urban fabric, transportation infrastructure, cultural composition, or the presence of alternative healthcare facilities, may vary smoothly across postal codes in ways not fully captured by our variable of interest. Importantly, Moran's I on the neighborhood-level residuals remained low and non-significant for all CTAS levels, confirming that the combination of fixed effects and postal-code random intercepts adequately accounts for spatial dependence at the analytical unit of interest. The spatial correlation in the random effects thus points to potential avenues for future research. These results have been added to the Results section:

*“Residual spatial autocorrelation (Moran’s I) was below 0.1 and non-significant in all models, while spatial autocorrelation in the postal-code random intercepts was significant for CTAS 3 and 4* but below 0.3 (0.23 and 0.16 respectively).”*

The corresponding values are reported in Table 2. We added in the discussion, in the paragraph about future work:

“*Significant spatial autocorrelation in the postal-code random intercepts found in our study suggest that unmeasured spatially structured factors may contribute to variation in low-acuity PED use beyond the determinants considered here. Future studies should consider larger scale measurements, such as differences in transportation infrastructure, cultural composition, or the presence of alternative healthcare facilities.*”
